# Advances in using MRI probes and sensors for *in vivo* cell tracking as applied to regenerative medicine

**DOI:** 10.1242/dmm.018499

**Published:** 2015-04

**Authors:** Amit K. Srivastava, Deepak K. Kadayakkara, Amnon Bar-Shir, Assaf A. Gilad, Michael T. McMahon, Jeff W. M. Bulte

**Affiliations:** 1 Russell H. Morgan Department of Radiology and Radiological Science, Division of MR Research, The Johns Hopkins University School of Medicine, Baltimore, MD 21205, USA.; 2 Cellular Imaging Section and Vascular Biology Program, Institute for Cell Engineering, The Johns Hopkins University School of Medicine, Baltimore, MD 21205, USA.; 3 Department of Oncology, The Johns Hopkins University School of Medicine, Baltimore, MD 21205, USA.; 4 F. M. Kirby Research Center for Functional Brain Imaging, Kennedy Krieger Institute, Baltimore, MD 21205, USA.; 5 Department of Chemical & Biomolecular Engineering, The Johns Hopkins University School of Medicine, Baltimore, MD 21205, USA.; 6 Department of Biomedical Engineering, The Johns Hopkins University School of Medicine, Baltimore, MD 21205, USA.

**Keywords:** Regenerative medicine, Stem cells, Magnetic resonance imaging, Paramagnetic contrast agents, CEST, Perfluorocarbon particles, Biosensor, Cell labeling, Cellular function

## Abstract

The field of molecular and cellular imaging allows molecules and cells to be visualized *in vivo* non-invasively. It has uses not only as a research tool but in clinical settings as well, for example in monitoring cell-based regenerative therapies, in which cells are transplanted to replace degenerating or damaged tissues, or to restore a physiological function. The success of such cell-based therapies depends on several critical issues, including the route and accuracy of cell transplantation, the fate of cells after transplantation, and the interaction of engrafted cells with the host microenvironment. To assess these issues, it is necessary to monitor transplanted cells non-invasively in real-time. Magnetic resonance imaging (MRI) is a tool uniquely suited to this task, given its ability to image deep inside tissue with high temporal resolution and sensitivity. Extraordinary efforts have recently been made to improve cellular MRI as applied to regenerative medicine, by developing more advanced contrast agents for use as probes and sensors. These advances enable the non-invasive monitoring of cell fate and, more recently, that of the different cellular functions of living cells, such as their enzymatic activity and gene expression, as well as their time point of cell death. We present here a review of recent advancements in the development of these probes and sensors, and of their functioning, applications and limitations.

## Introduction

Magnetic resonance imaging (MRI; see Box 1 for a brief history on its development) is a non-invasive imaging technique that allows the visualization of the internal structures of the body in health and disease; it has thus been used as a diagnostic tool, with a wide range of medical applications, for more than 30 years. The principle of MRI is based on manipulating the magnetic properties of the protons and neutrons contained in atomic nuclei present in the body (most commonly, those found in the atoms of hydrogen). The motion of these nuclei produces a small magnetic moment (see Box 2 for a glossary of terms). When a body is placed in the magnetic field of the MRI scanner, the magnetic moment of these nuclei aligns with the direction of the magnetic field. A radiofrequency (RF) pulse is then applied to the body in the scanner, which excites the nuclei such that there are transitions between lower and higher energy spin states. Once the RF pulse is given, the nuclei return to their equilibrium state (a process called relaxation), releasing their absorbed extra energy and emitting an RF signal. This signal is detected by the scanner’s RF coils and is then used to generate a detailed image of the body’s tissues. By using MRI contrast agents (see the following sections, Boxes 2 and 3, and Table 1 for more details), the contrast of this image, and so the visibility of specific body structures, can be improved. Subsequent advancements in this field made it possible to use these contrast agents to label specific cell types and thus to monitor cells at the molecular level.

**Table 1.**
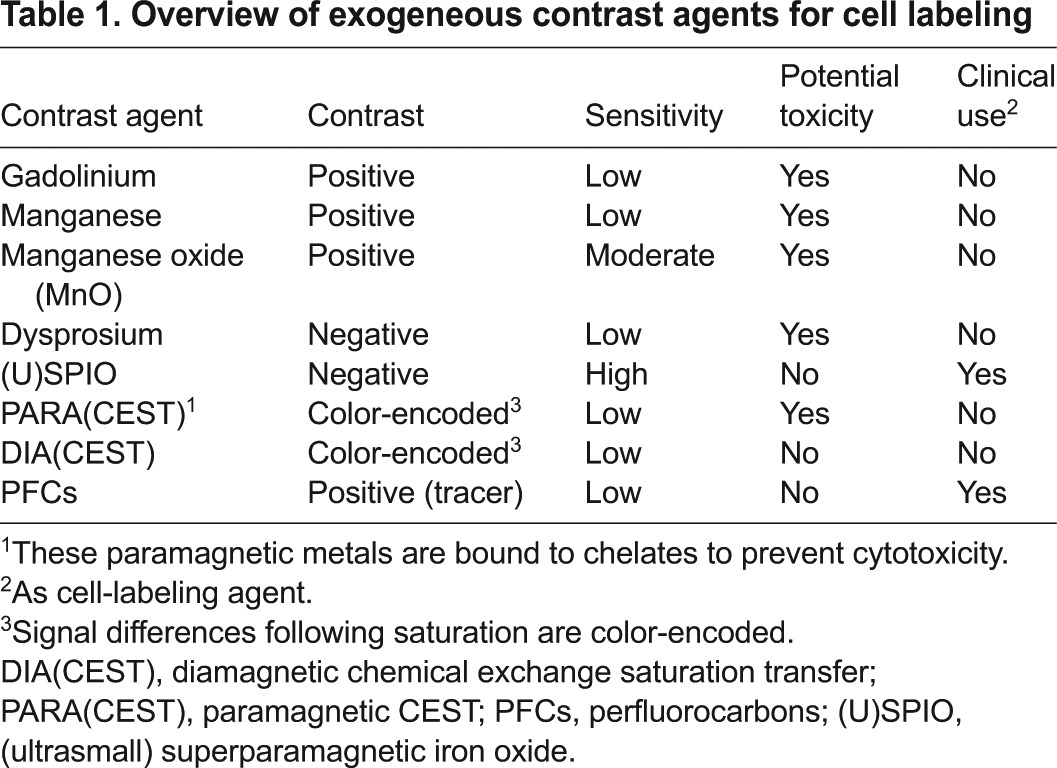
Overview of exogeneous contrast agents for cell labeling

**Box 1. A brief history of MRI**

The phenomenon of nuclear magnetic resonance (NMR) was described for the first time in 1946 by Bloch and Purcell (Bloch et al., 1946; Purcell et al., 1946), who later shared the 1952 Nobel Prize in Physics. A few years later the key discovery that nuclei in chemically distinct sites in the same molecule resonate at slightly different frequencies was reported (Dickinson, 1950; Proctor and Yu, 1950), which led to the important application of NMR in the field of analytical spectroscopy. In 1971, Damadian recognized the diagnostic potential of NMR to discriminate between different tissues (Damadian, 1971). In 1973, the first magnetic resonance (MR) image was published by Lauterbur (Lauterbur, 1973), who, together with Peter Mansfield, received the 2003 Nobel Prize in Medicine or Physiology. It was realized that, because the resonance frequency is proportional to the strength of the applied magnetic field, a magnetic field gradient would give rise to a range of resonance frequencies, which reflect the spatial distribution of protons, for example those in water (the most abundant molecule in cells). A cross-sectional image of a living mouse was published in January 1974 (Lauterbur, 1974) and, a few years later, paramagnetic manganese was used as the first ‘MR contrast agent’ (Lauterbur et al., 1978). After further imaging-reconstruction techniques employing back-projection (Hounsfield, 1973) or two-dimensional Fourier analysis (Kumar et al., 1975), together with the emergence of sufficiently fast and powerful computers, human magnetic resonance imaging (MRI) made its entrance in 1977–1978. After Bottomley et al. built the first high-field [1.5 Tesla (T)] whole-body MRI/MRS scanner (Bottomley et al., 1983), MRI became a ubiquitous tool in clinical diagnosis, revolutionizing medicine, with over 25,000 of these systems in use today.

**Box 2. A glossary of terms**

CEST: chemical exchange saturation transfer; an MRI technique that creates contrast for selected protons present in specific chemical groups.
Chelate: a cage-like molecule that binds and protects potentially toxic metal ions (such as gadolinium).
Chemical shift: a change in the resonance frequency of protons away from that of water protons, usually expressed as ppm (parts per million).
Contrast agent: a chemical compound that alters proton relaxation times, creating a different MRI signal.
Electroporation: the process of making a cell membrane temporarily permeable, using electrical currents, in order to allow added contrast agents to go into the cell.
Gadolinium: a rare earth metal or lanthanide element that contains seven unpaired electrons and is clinically, in chelated form, the most widely used contrast agent.
iCEST: ion chemical exchange saturation transfer; a ^19^F MRI technique to detect a specific metal ion using a fluorinated probe.
Lanthanides: rare earth metals in the group of the periodic table numbered 57–71 with a paramagnetic moment that shortens the relaxation time and/or shifts the resonance frequency of protons in water.
Magnetic moment: the magnetic moment of an atomic nucleus arises from the spin of the protons and neutrons. It is mainly a magnetic dipole moment and, if large enough, can shorten the T2 relaxation time.
Magnetodendrimer: a SPIO contrast agent coated with dendrimers as a prototype for the development of SPIO-bound transfection agents for universal cell labeling of non-phagocytic cells.
miCEST: multi-ion chemical exchange saturation transfer; a ^19^F MRI technique to detect specific metal ions separately and simultaneously using a single fluorinated probe.
NMR: nuclear magnetic resonance; a physical phenomenon in which nuclei (commonly protons) in a magnetic field absorb and re-emit electromagnetic radiation.
PARACEST: paramagnetic chemical exchange saturation transfer; an MRI technique that creates contrast for protons present nearby certain lanthanide metal ions.
PET: positron emission tomography; an imaging technique that detects pairs of gamma rays emitted indirectly by a positron-emitting radionuclide (tracer), which is introduced into the body on a biologically active molecule.
Relaxation: the process of returning to equilibrium (base level) for excited protons pulsed to a higher energy state; the time it takes to fall back to equilibrium is related to the relaxation time.
Relaxation time: the time it takes for a proton to return from its excited state to its original state. There are three relaxation times: T1 or longitudinal relaxation time, T2 or transverse relaxation time, and T2*, which is T2 without rephasing (single T2 decay).
Relaxivity: the ability of an agent to increase the relaxation of protons in water.
RF pulse: radiofrequency pulse; a wave that excites protons so that they can provide an MRI signal (conventional MRI) or that cancels their ability to create an MRI signal (CEST MRI).
(U)SPIO: (ultrasmall) superparamagnetic iron oxide; a magnetic nanoparticle MR contrast agent that makes labeled cells appear dark on imaging.

**Box 3. A brief introduction to contrast agents**

The so-called ‘T1 agents’ primarily affect the T1 (spin-lattice) relaxation time of a tissue and create hyperintense contrast (brightening the tissue of interest). Commonly, they are referred to as paramagnetic agents, which have one or more unpaired electrons. They behave as a spinning dipole in a static magnetic field, creating miniscule local field alterations. This affects directly the magnetization of the surrounding protons, leading to shorter T1 relaxation times. Gadolinium, with its seven unpaired electrons, is the most effective agent and is clinically widely used. Superparamagnetic iron oxide (SPIO) particles and dysprosium agents are referred to as ‘T2 agents’, which create much stronger alterations in the local magnetic field, causing the protons to go out of phase (affecting largely the T2 spin-spin relaxation time); this results in the tissue of interest generating less signal, becoming hypointense compared to the surrounding non-targeted tissue. Chemical exchange saturation transfer (CEST) magnetic resonance (MR) contrast agents create differences in proton signal when frequency-selective saturation of their exchangeable protons is applied. These agents are based on amino acids, proteins and sugars, and, because they are devoid of metals, are often referred to as diamagnetic or DIACEST agents. Paramagnetic CEST (PARACEST) agents also create contrast based on the CEST principle (see Box 2). The difference is that they contain paramagnetic metals with a high magnetic moment that do not affect T1 but instead induce large chemical shifts from the proton resonance frequency. Finally, ^19^F agents, such as perfluorocarbons, are technically not ‘contrast’ agents (because there is no background signal to contrast with) and can be referred to as MRI ‘tracers’ (Holland et al., 1977), analogous to those used in nuclear medicine.

The application of MRI to cellular imaging *in vivo* has proved particularly useful in the field of regenerative medicine research, where it allows the tracking of engrafted cells and the monitoring of their physiological responses in a non-invasive manner. Over the past two decades, stem cells have been increasingly used as potential therapies for different disease conditions, particularly those in which cell replacement can restore the normal function of tissue or organs subsequent to their damage or degeneration. For example, as reported in the NIH public clinical trials database (http://www.clinicaltrials.gov; accessed 26 January, 2015; only open studies included, unknown status excluded), 1502 clinical trials at different phases are currently using stem-cell-based therapies to treat various disease conditions, e.g. myocardial infarct, neurodegenerative diseases and autoimmune diseases.

Based on the increasing numbers of cell-replacement therapies, it has become imperative to monitor non-invasively the engraftment of cells *in vivo* to determine the overall safety and efficacy of these approaches. For example, two FDA-approved cord blood products, ‘Hemacord’ (manufactured by New York Blood Center, Inc.; www.fda.gov; Submission Tracking Number: BL 125397/0) and ‘HPC-Cord Blood’ (manufactured by Clinimmune Labs, University of Colorado Cord Blood Bank; www.fda.gov; Submission Tracking Number: BL 125391/0) are being used for hematopoietic stem cell replacement therapies. Both cell therapies are systemically delivered, non-specific, and rely on the engraftment of an extremely large number of cells (recommended minimum dose: 2.5×10^7^ nucleated cells/kg body weight), with the assumption that enough cells will find their way to the target sites. Only non-invasive imaging renders it possible to evaluate the homing of such cells *in vivo*, thereby confirming the efficacy of such a therapeutic approach.

In certain conditions, such as in neurodegenerative disorders and in type 1 diabetes, poor engrafted cell survival, due to a hostile host tissue microenvironment or to inadequate nutrient support (Robberecht and Philips, 2013; Thomas et al., 2013), is a major barrier to the success of stem-cell-based therapies. Thus, the capacity to determine, in real time, the physiological state of engrafted cells, in terms of their gene expression and enzymatic activity, and to monitor their cell survival, will enable clinicians to better assess the therapeutic outcome of such treatments. This might, in turn, lead to the development of more-effective therapies.

MRI is an ideal tool for the *in vivo* tracking and ‘sensing’ of engrafted cells because of its ability to image deep inside tissue and to gather accurate anatomical and physiological information with high temporal resolution and sensitivity (Srivastava and Bulte, 2014). MRI could also be used to monitor alterations in cell function, tissue damage and changes in the dynamics of the biological processes that are associated with certain diseases (Haris et al., 2014; Yoo and Pagel, 2006). This use of MRI for non-invasive cell tracking first emerged from the use of MRI to label immune cells (Bulte et al., 1992; Bulte et al., 1993), and was followed by the first clinical application of MRI cell tracking to label and follow the fate of anti-tumor dendritic cells, used as cancer vaccines (de Vries et al., 2005).

In recent years, great progress has been made in the development of novel MRI sensors to monitor the different cellular functions of engrafted cells. In this Special Article, we describe recent advances in the development of MRI probes and sensors that are used for cell tracking and for detecting cellular functions *in vivo*. We also discuss the limitations of these approaches, their associated safety issues and the opportunities for their further improvement.

**Following the cell: MR contrast agents**

Contrast agents (see Table 1) are exogenous compounds that are administered intravenously or orally prior to MRI in order to improve the visibility of body structures for clinical diagnostic purposes. These agents change the relaxation rate of protons in water, creating a change in signal on MRI (see Box 3). However, contrast agents can also be used to pre-label cells *ex vivo* before transplantation, which is the most commonly used approach in MRI-based cell tracking.

There are different ways to incorporate contrast agents into living cells, such as by, for example, the use of transfection agents (Frank et al., 2002) and the use of translocation peptides. In this section, we discuss the main types of magnetic resonance (MR) contrast agents, how they function and their applications in clinical settings, as well as in experimental cell-tracking and regenerative approaches.

### Paramagnetic gadolinium agents

Paramagnetic MR contrast agents (Table 1) are widely used in clinical MRI. Gadolinium (III) (Gd^3+^) chelates (see Box 2) are the most effective paramagnetic contrast agents, owing to their seven unpaired electrons. The unpaired electrons of Gd^3+^ create a magnetic moment that increases the T1 of the surrounding water proton spins, creating ‘positive’ contrast on a T1-weighted scan (see Box 3). As a research tool, Gd^3+^ has been used to label and track different types of stem cells, such as hematopoietic progenitor cells, monocytic cells, endothelial progenitor cells and mesenchymal stem cells in cell transplantation studies in small animals (Agudelo et al., 2012; Guenoun et al., 2012; Hedlund et al., 2011). Because they are not nanoparticles, the cellular uptake of Gd^3+^ chelates occurs by pinocytosis (a non-specific form of endocytosis in which small particles present in the extracellular fluid are internalized into cells) or via electroporation (see Box 2). However, overall, the low sensitivity of these contrast agents and their low uptake by cells is one of the main barriers to cell labeling with Gd^3+^. Different methods, e.g. transfection using transfection agents (lipofectin and lipofectamine) (Rudelius et al., 2003) or coupling of the contrast agent to a membrane-translocation peptide (13-mer HIV-tat peptide) (Bhorade et al., 2000), can be used to increase the uptake of paramagnetic chelates. In the last few years, several advanced paramagnetic contrast agents with improved uptake efficiency, e.g. Gd hexanedione, liposomal-based Gd nanoparticles, and gadofluorine (Nejadnik et al., 2012b), have been developed and successfully used for cellular imaging (Ghaghada et al., 2009; Tseng et al., 2010).

However, the major limitation to the use of Gd^3+^ contrast agents is the long-term presence of Gd^3+^ in cells, which might lead to toxicity, either to the cells themselves or to the surrounding host tissue. This has been reported in the case of Gd^3+^ chelates that are not rapidly cleared in patients with impaired kidney function, causing severe fibrosis and even death (Thomsen, 2006). In addition, in stroke models, as for example in a rat model of middle cerebral artery occlusion (MCAO), transplantation of neural stem cells labeled with gadolinium-rhodamine dextran (GRID) did not significantly improve the therapeutic outcome. To the contrary, T2-weighted MRI of MCAO rats monitored for 1 year revealed that the presence of GRID-labeled cells slightly increased the infarct size in the treated rats when compared with MCAO rats that were not treated with cells, whereas the lesion size was decreased by 35% in animals that were treated with cells that did not have gadolinium labeling (Modo et al., 2009). The exact mechanism of gadolinium toxicity is not known, but dechelated (free) gadolinium is believed to be taken up in the bone marrow, where it might interfere with metal ion hemostasis of bone marrow stem cells.

A different class of paramagnetic contrast agents is represented by the so-called PARACEST (paramagnetic chemical exchange saturation transfer) agents (see Boxes 2, 3; Table 1) (Zhang et al., 2001). Because different metals induce different chemical shifts (see Box 2 for a glossary of terms) in resonance frequencies, PARACEST MRI can be used to track two different types of cell populations simultaneously by labeling them with two different PARACEST agents (Aime et al., 2005). As further proof-of-principle, the two PARACEST agents Yb- and Eu-HPDO3A have been successfully used *in vivo* to track two cell populations (macrophages and melanoma cells) (Ferrauto et al., 2013). One of the limitations of PARACEST agents, however, is their intrinsically low contrast sensitivity; to detect sufficient contrast typically requires concentrations ranging from 1 to 10 mM. At such concentrations, these paramagnetic metal agents might become toxic when present in the body for prolonged periods, as in the case for Gd^3+^ described above.

### Paramagnetic manganese agents

In addition to Gd^3+^, manganese (Mn II) is another potentially useful positive contrast agent for T1-weighted MRI (see Box 3 and Table 1). The kinetics and behavior of Mn^2+^ ions in the cell mimic those of calcium (Ca^2+^) ions, because Mn^2+^ ions enter cells through ligand- or voltage-gated Ca^2+^ ion channels (Narita et al., 1990). Therefore, Mn^2+^-enhanced MRI (MEMRI) has been used to study neuronal activity and to visualize neuronal connectivity in different animal models (Silva and Bock, 2008). Few reports exist concerning the use of manganese-based contrast agents in cell tracking. One study used MnCl_2_ as a contrast agent for the labeling of lymphocytes as proof-of-principle (Aoki et al., 2006), but no *in vivo* studies using this approach have been performed. By contrast, manganese oxide (MnO) nanoparticles have been used as an MR contrast agent to detect cells *in vivo.* One of the advantages of using these nanoparticles *in vivo* is that they can be combined with superparamagnetic iron oxide (SPIO; discussed below, and see Box 2) particles to detect two cell populations with differential cell contrast (positive versus negative) simultaneously (Gilad et al., 2008a). More recently, silica-coated MnO nanoparticles have been developed as a positive T1 contrast agent for the labeling and MR tracking of mesenchymal stem cells (Kim et al., 2011).

### Superparamagnetic agents

**Fig. 1.**
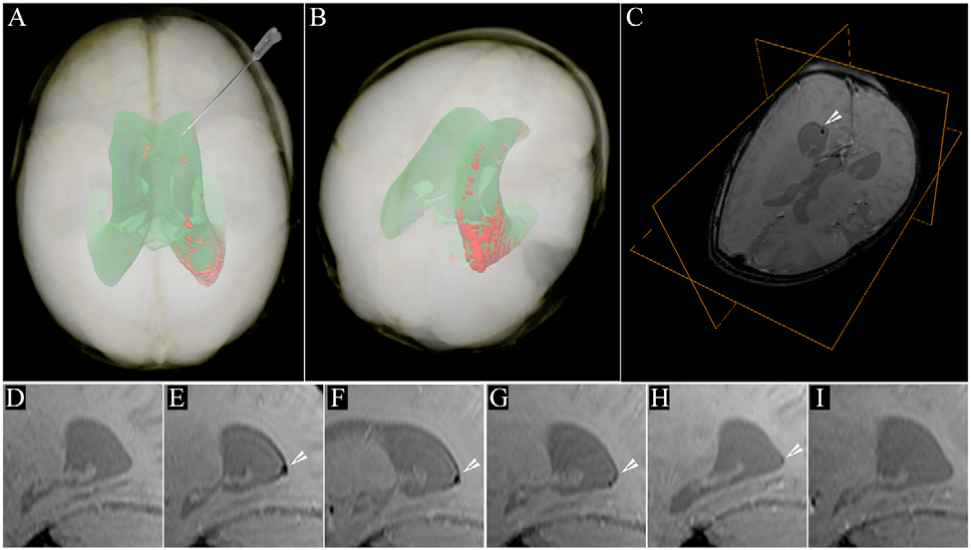
**MRI of SPIO-labeled cells in a human brain.** MRI evaluation of long-term tracking of transplanted cells labeled with superparamagnetic iron oxide (SPIO) nanoparticles in the human brain. SPIO-labeled autologous cord-blood-derived cells were transplanted in the frontal horn of the lateral ventricle of a patient with global cerebral ischemia. (A) 24 hours post-transplantation (PT) the distribution of the SPIO signal generated from the transplanted cells is detectable within the occipital horn of the right lateral ventricle (red); the projection of the ventricular system is shown in green. The needle indicates the route and trajectory of cell transplantation via the frontal horn. This is a volume rendering of MRI data of the patient’s head. (B) Location of the hypointense SPIO signal generated from transplanted cells in a postero-superior view of the patient’s head, where it can be better appreciated. (C) T2*-weighted image of the patient’s head with an orthogonal view centered on the cellular SPIO signal in the occipital horn (arrowhead). (D-I) The longitudinal dispersion of the cellular SPIO signal within the occipital horn (arrowheads) is shown in sagittal T2*-weighted MRI scans at different time points: (D) pre-transplantation; (E) 24 hours PT; (F) 7 days PT; (G) 2 months PT; (H) 4 months PT; and (I) 33 months PT. Figure and data reproduced with permission (Janowski et al., 2014).

SPIO nanoparticles have a much stronger MR relaxivity and higher sensitivity compared to paramagnetic agents (see Table 1), which makes them generate an MRI signal that is strong enough to visualize a small number of cells (Fig. 1). The presence of thousands to millions of magnetically aligned iron atoms in the SPIO core enables sensitive detection after the cells internalize these nanoparticles. Iron particle size varies from 10–50 nm [very small or ultra-small SPIO (VSOP and USPIO, respectively)] to >1 μm [micrometer-sized iron oxide (MPIO)] (Bulte and Kraitchman, 2004). SPIOs were first utilized to label and track transplanted cells in the rat brain (Hawrylak et al., 1993; Norman et al., 1992). Over the years, iron oxide particles have been developed that have higher relaxivities and more efficient intracellular labeling (Nkansah et al., 2011; Tang and Shapiro, 2011). Non-phagocytic cells cannot internalize SPIO nanoparticles; the most commonly used approach to label such cells is through the use of transfection agents, as originally demonstrated for magnetodendrimers (see Box 2 for more details) (Bulte et al., 2001).

As an example of their clinical use, SPIO nanoparticles have been used to track, using MRI, the homing of injected mesenchymal stem cells to the central nervous system (CNS) of individuals with multiple sclerosis (MS) and amyotrophic lateral sclerosis (ALS) (Karussis et al., 2010). This approach is particularly valuable because SPIO nanoparticle labeling does not affect the differentiation capacity (Nejadnik et al., 2012a; Wang et al., 2011), viability or function (Richards et al., 2012) of transplanted stem cells, except for chondrogenesis by mesenchymal stem cells, where it interferes with forming the extracellular matrix of cartilage (Bulte et al., 2004; Kostura et al., 2004).

In the past, only two clinical-grade, iron-oxide-based agents have been developed and approved for MRI of the liver: (1) SPIO nanoparticles coated with dextran as a polysaccharide (i.e. ferumoxides, also known as Endorem^®^ in Europe and Feridex^®^ in the USA) and (2) SPIO nanoparticles coated with low molecular weight carboxydextran (i.e. ferucarbotran, also known as Resovist^®^). Although these SPIO formulations have been used for several clinical studies in the past, they are no longer manufactured because of economic considerations (Bulte, 2009). The agents never sold well for their FDA-approved application (i.e. detection of liver tumors) and, over time, abdominal MRI techniques have become more advanced to detect those masses without the need of administering contrast.

A limitation to the use of SPIO nanoparticles is their occasional extracellular deposition in tissues, either by active exocytosis (Cromer Berman et al., 2013) or passive release through the death of transplanted cells (Terrovitis et al., 2008). Deposited iron particles are scavenged by macrophages, which can then generate a false signal on MRI. As a result, the clinical application of these nanoparticles is limited to their short-term use and includes the MR-guided delivery of cells in real time (Barnett et al., 2007; Kraitchman et al., 2003) and the subsequent immediate monitoring of their homing and engraftment. As such, the use of these iron nanoparticles is restricted to assessing the acute retention of labeled cells and their short-term distribution in the body.

### Fluorinated agents

Fluorine (^19^F) is the naturally abundant isotope of fluorine, with a nuclear magnetic resonance (NMR; see Boxes 1, 2) sensitivity of 83% compared with hydrogen (^1^H). It is also not radioactive, in contrast to the ^18^F isotope, which is used in positron emission tomography (PET; see Box 2) imaging. ^19^F MRI has emerged as a novel technology for not only tracking transplanted immune cells, but also endogenous macrophage-type cells that are present in several inflammatory disorders, such as autoimmune myocarditis and inflammatory bowel disease (Ahrens and Bulte, 2013; Ahrens and Zhong, 2013; Temme et al., 2012). Although SPIO nanoparticles are currently the most commonly used agent for MRI cell tracking (Cromer Berman et al., 2011), the relationship between the contrast, free iron and the concentration of SPIO is non-linear, and, therefore, it is difficult to quantify (Lebel et al., 2006; Rad et al., 2007). In addition, the SPIO-induced negative contrast is not truly specific and could be difficult to interpret at times, particularly when other sources of low signal contrast are present, as occurs for example in blood vessels or when a patient is bleeding – following illness or injury – where blood iron accumulates and is converted to a negative iron oxide contrast agent. By contrast, ^19^F MRI provides a more accurate, unambiguous detection of labeled cells (given the lack of background signal). Moreover, the relationship between the concentration of the ^19^F and signal intensity is directly proportional and linear over a wide range of concentrations, and the signal can be quantified directly from the acquired images (Srinivas et al., 2007). The lack of a detectable background in the ^19^F signal in biological tissues leads to higher visibility of the target cells, much like ‘hot spots’ emerging from an empty background (Bulte, 2005).

**Fig. 2.**
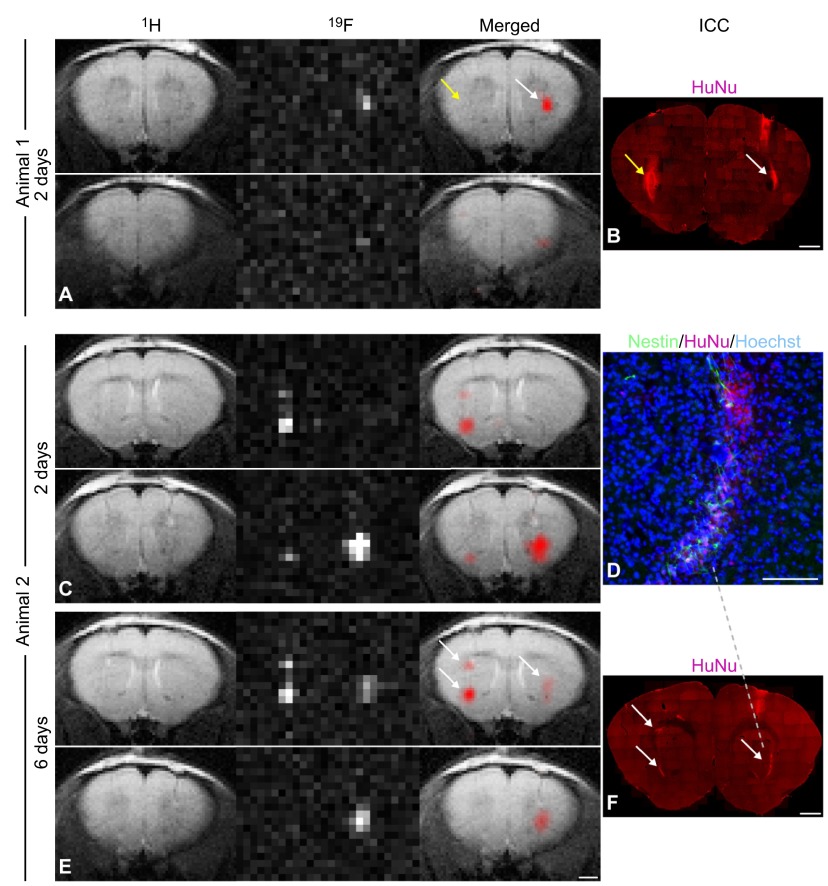
**MRI of ^19^F-labeled cells in mice.** MRI tracking of human neural stem cells (NSCs) labeled with fluorine (^19^F) after implantation in the mouse striatum. Animal 1: (A) ^1^H, ^19^F and merged MR images of a mouse brain acquired 2 days after injection with non-labeled control cells into the left striatum (yellow arrow) and with ^19^F-labeled NSCs into the right hemisphere (white arrow). Shown are two sequential slices demonstrating that only the labeled cells generated a ^19^F signal. (B) Human nuclear antigen (HuNu) staining was used to confirm the presence of NSC grafts in both hemispheres (yellow and white arrows). Animal 2: comparison of MR images of another mouse brain acquired (C) 2 days and (E) 6 days after grafting with human NSCs, showing no major signal loss in the ^19^F images over time. Shown are two sequential slices. This animal received two deposits of ^19^F-labeled cells in the left striatum and one deposit in the right striatum (E, arrows); the high spatial resolution of ^19^F MRI allows the two cell clusters in the left hemisphere to be clearly distinguished, demonstrating the potential of this technique for the detection of small numbers of cells *in vivo* within a restricted area. (D) Implantation of NCSs was confirmed histologically via colocalization of HuNu, the stem-cell marker nestin and the nuclear marker Hoechst in the mouse striatum. (F) HuNu staining correlated with the location and intensity of ^19^F signal generated from cell clusters (arrows). Scale bars: 50 μm for D; 1 mm for all others. Figure and data reproduced with permission (Boehm-Sturm et al., 2011).

Perfluorocarbons (PFCs), which have many fluorine atoms with identical chemical shifts, are most commonly used for ^19^F MRI cell tracking applications, and they include perfluoro-15-crown-5-ether, linear perfluoropolyethers and perfluorooctyl bromide (Kadayakkara et al., 2014; Ruiz-Cabello et al., 2011). PFCs, being lipophobic and hydrophobic, are formulated into stable nanoemulsions by high-energy sonication for cell labeling (Janjic and Ahrens, 2009). Similar to SPIO-labeled cells, cells are labeled with PFC nanoemulsions *ex vivo* and then injected into the body (Ahrens and Zhong, 2013). Of note is that PFCs with different chemical shifts can be used to visualize two distinct populations of cells simultaneously *in vivo* (Partlow et al., 2007). As for regenerative medicine, neural stem cells have been successfully labeled with PFCs, and then visualized following their implantation into the brain (Fig. 2) (Bible et al., 2012; Boehm-Sturm et al., 2011; Ruiz-Cabello et al., 2008). Previous studies have shown that PFC labeling has a minimal effect on cell viability and cellular functions, such as differentiation and proliferation, both *in vitro* and *in vivo* (Boehm-Sturm et al., 2011; Ruiz-Cabello et al., 2008; Barnett et al., 2011; Keupp et al., 2011; Partlow et al., 2007).

In addition to tracking cells, PFCs can be used to probe cell function by sensing intracellular oxygen tension. For example, one study combined cell-labeling technology with the oxygen-sensing ability of PFCs to measure the intracellular oxygen tension in brain tumor models (Kadayakkara et al., 2010; Zhong et al., 2013), based on the principle that O_2_ molecules change the ^19^F T1 relaxation time. This study reported that changes in oxygenation following tumor treatment with chemotherapy and immunotherapy provided a reliable imaging biomarker for evaluating the efficacy of these anti-tumor treatments. This approach could potentially be used to assess possible hypoxia at the site of stem cell transplantation, and to determine its effect on stem cell survival. The promising preclinical data and safety profile of PFCs make ^19^F MRI ready for clinical translation; recently, the feasibility of using ^19^F imaging of cells in a clinical setting has been demonstrated (Ahrens et al., 2014).

There are, however, limitations to the amount of PFCs that can be incorporated into cells: their size, similar to MPIOs, means that they take up a significant amount of cytoplasmic volume. Thus, the population number of cells to monitor should be above a certain threshold for detection, and that might vary depending on cell type. The minimum amount of cells that can be detected using ^19^F-based imaging ranges from 10^3^ to 10^5^ cells (Ahrens and Bulte, 2013). ^19^F MRI also requires the use of separate coils for image acquisition, or use of a multinuclear detector coil, neither of which is readily available in many MRI centers. However, the resonance frequency of ^19^F is close to that of ^1^H; therefore, existing ^1^H coils could be adapted for use as dual-tuned coils, capable of imaging both ^1^H and ^19^F nuclei.

### Highly-shifted proton MRI

**Fig. 3.**
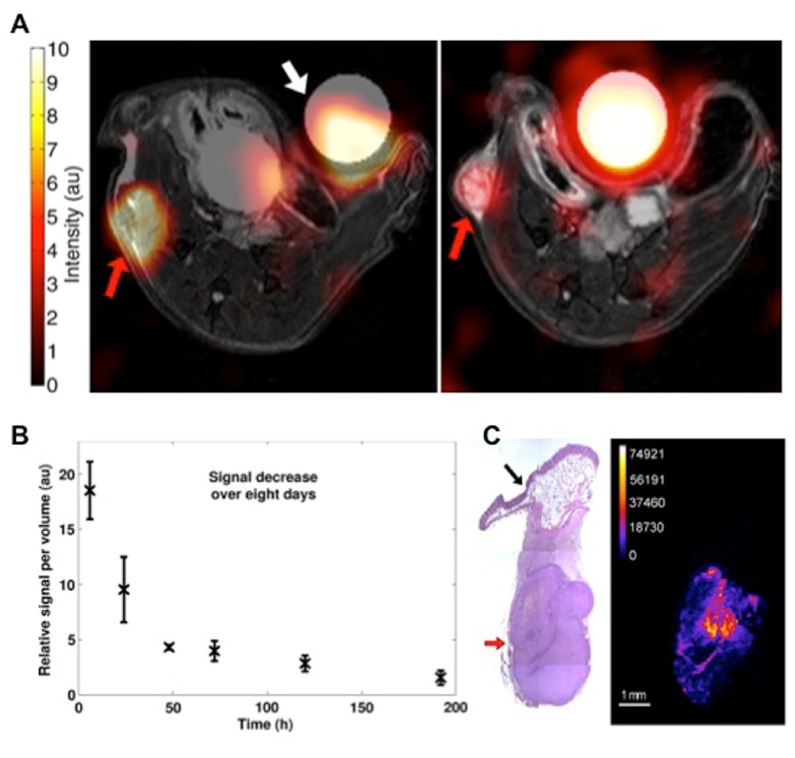
**MRI of highly shifted proton (HSP)-labeled cells in mice.** (A) Ultra-short echo time (UTE) image of a mouse subcutaneously implanted with Tm-DOTMA-labeled fibrosarcoma shows that these cells are detectable *in vivo* immediately (left) and 8 days after (right) injection. The ‘hot spot’ HSP signal is displayed superimposed over the anatomic ^1^H MRI T2-weighted images. In addition to the tumor (red arrow), a reference tube containing 0.25 mmol/l of aqueous Tm-DOTMA solution (white arrow) is shown. (B) The Tm-DOTMA signal in the tumor decreases with time, due to cell division. au, arbitrary units. (C) Corresponding mass spectrometric Tm image (right; color scale in arbitrary units) corresponds to tumor location on histology (left, red arrow), whereas surrounding fatty tissue (black arrow) showed no Tm signal. Reproduced with permission (Schmidt et al., 2014).

In 2014, a novel ‘hot spot’ highly shifted proton (HSP) MRI technique for cell tracking was described that directly detects dysprosium (Dy)- or thulium (Tm)- 1,4,7,10-tetraazacyclododecane-1,4,7,10-tetramethyl-1,4,7,10-tetraacetic acid (DOTMA)-labeled protons inside cells (Bulte, 2014; Schmidt et al., 2014). The principle behind HSP MRI is that certain lanthanides (see Box 2 for a glossary of terms), in particular Tm and Dy, cause a chemical shift of proton resonance frequency, one that is far away (100 ppm) from the water peak (from which the MRI signal is normally collected). When the MRI excitation pulse is applied at that chemical shift, only cells that contain the Dy- or Tm-bound protons will resonate and generate a signal. A customized pulse sequence, termed ultra-short echo time (UTE) MRI, was used to take advantage of the dramatically shortened T1 value of the protons bound to the chelate (Schmidt et al., 2014). When human fibrosarcoma tumor cells were labeled with Tm-DOTMA by electroporation and implanted into the flank of nude mice, the tumor could clearly be detected as a ‘hot spot’ on HSP MRI, and the overlay with conventional T2-weighted MRI allowed its anatomical localization (Fig. 3). HSP MRI is thus somewhat comparable to ^19^F MRI, and has a lower approximate cell detection number of 1×10^4^ cells.

## Beyond cell tracking: determining cellular function with MRI

Beyond cell tracking, MRI now has the added potential of being used to monitor enzymatic activity, gene expression, metal ion homeostasis and cell death *in vivo*. In this section, we discuss the most recent advances in the development of MRI probes and sensors to monitor cellular function, and also discuss the potential applications of these approaches to regenerative medicine.

### Monitoring enzymatic activity: use of sensors

In several preclinical studies, stem cells have been genetically modified to overexpress growth factors and enzymes in order to enhance their therapeutic properties in different disease conditions (Li et al., 2014; Krakora et al., 2013; Liang et al., 2013a; Suzuki et al., 2008; Xie et al., 2007). However, changes in the expression or level of activity of endogenous enzymes can also be associated with different diseases, requiring imaging methods to be developed that can distinguish between the two conditions.

**Fig. 4.**
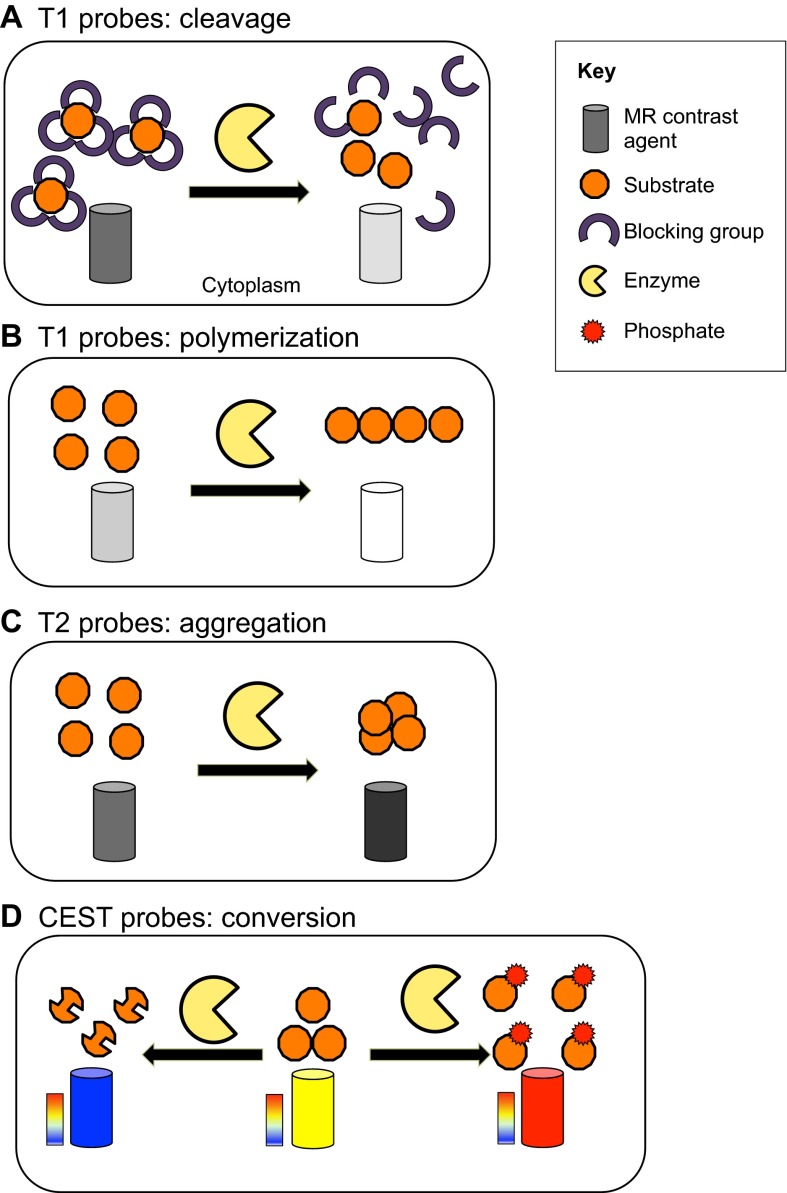
**Schematic depicting the different mechanisms of enzyme-induced changes in contrast.** (A) T1 (positive) contrast can be increased by enzymatic removal of a blocking group (Louie et al., 2000). (B) T1 (positive) contrast can be increased by enzymatic polymerization, where the polymerized contrast agent has a higher relaxivity than the single molecules (Chen et al., 2004). (C) T2 (negative) contrast can be increased by enzymatic cleavage/conversion of superparamagnetic iron oxide (SPIO) agent surface groups, leading to particle aggregation (Schellenberger et al., 2008). (D) Chemical exchange saturation transfer (CEST) contrast can be reduced (blue) by enzymatic cleavage (removal) of the exchangeable proton (Liu et al., 2011) or increased (red) by accumulation of the probe in the cell following enzymatic phosphorylation (Bar-Shir et al., 2013c). Vertical color bar represents CEST contrast in color-coded hot scale.

In recent years, several MRI-based sensors have been designed to measure the expression and activity of both endogenous and recombinant enzymes. Most of these studies have been performed *in vitro* as proof-of-principle, with few *in vivo* examples as of yet. These sensors can either be substrates of the enzyme of interest conjugated to a contrast agent, or a substrate that changes its chemical exchange saturation transfer (CEST; see Boxes 2, 3) properties upon enzymatic conversion (Fig. 4). The contrast of the MRI signal is then either decreased or increased when the enzyme is present. The enzyme’s substrate can generate T1-weighted (Chen et al., 2004; Duimstra et al., 2005; Louie et al., 2000), T2-weighted (Westmeyer et al., 2010), T_2_*-weighted (Hingorani et al., 2013; Liu et al., 2011; Schellenberger et al., 2008), ^19^F (Matsuo et al., 2013) or CEST (Airan et al., 2012; Liu et al., 2011) MRI-based contrast.

β-galactosidase (β-gal; encoded by the *lacZ* reporter gene) is a commonly used enzyme for the detection of transplanted cells by histology. Here, the enzyme converts its substrate, X-gal, to a dense dark-blue-colored product that can be readily detected with conventional light microscopy. Hence, it is not surprising that it has been a widely used platform for the development of MRI-based probes to detect enzymatic activity. As demonstrated early on by Louie et al. (Louie et al., 2000), a galactose-blocked contrast agent can be unblocked by cleavage by cells expressing recombinant β- gal, ‘opening up’ the contrast agent to the nearby protons in water and making it a more effective T1 contrast. Mason and co-workers developed libraries of ^19^F-modified substrates for monitoring β-gal activity with ^19^F magnetic resonance spectroscopy (MRS) and MRI (Cui et al., 2004; Kodibagkar et al., 2006; Liu et al., 2007; Yu et al., 2012). Other enzymes (either endogenous or genetically engineered) that have been used as targets for responsive MRI probes include: creatine kinase (Haris et al., 2014), matrix metalloproteinase-2 (MMP-2) (Catanzaro et al., 2013; Matsuo et al., 2013), matrix metalloproteinase-9 (MMP-9) (Schellenberger et al., 2008), myeloperoxidase (MPO) (Rodríguez et al., 2010), nitroreductase (Matsuo et al., 2013), urokinase plasminogen activator (uPA) (Yoo et al., 2013), alkaline phosphatase (Westmeyer et al., 2010), cytosine deaminase (Liu et al., 2011), β-glucuronidase (Duimstra et al., 2005), acetylCoA synthetase (Bastiaansen et al., 2013), carboxypeptidase G2 (Jamin et al., 2013; Jamin et al., 2009), transglutaminases (Tei et al., 2010), protein kinase A (PKA) (Airan et al., 2012; Shapiro et al., 2009), caspase-3 (Perez et al., 2002) and others (Razgulin et al., 2011) (see Table 2). Many of these enzymes are associated with different disorders and can serve as disease biomarkers. For instance, a change in the level of MMP can lead to disorders of the growth plate and can contribute to altered skeletal development, to cardiovascular disease, arthritis, cancer and CNS disease (Malemud, 2006). MPO can serve as a predictor for myocardial damage (Brennan et al., 2003) and uPA as a biomarker for pancreatic tumor invasion and metastasis (Yoo et al., 2013). Similarly, creatine chemical exchange saturation transfer (CrEST) MRI can map deficiencies in metabolites produced by necrotic myocardium and detect ischemic myocardium associated with early-stage heart disease (Haris et al., 2014). Non-invasive imaging of these enzymes could help to both predict disease onset and monitor its progression, but could also aid in the development of cell therapies with higher therapeutic benefit.

**Table 2.**
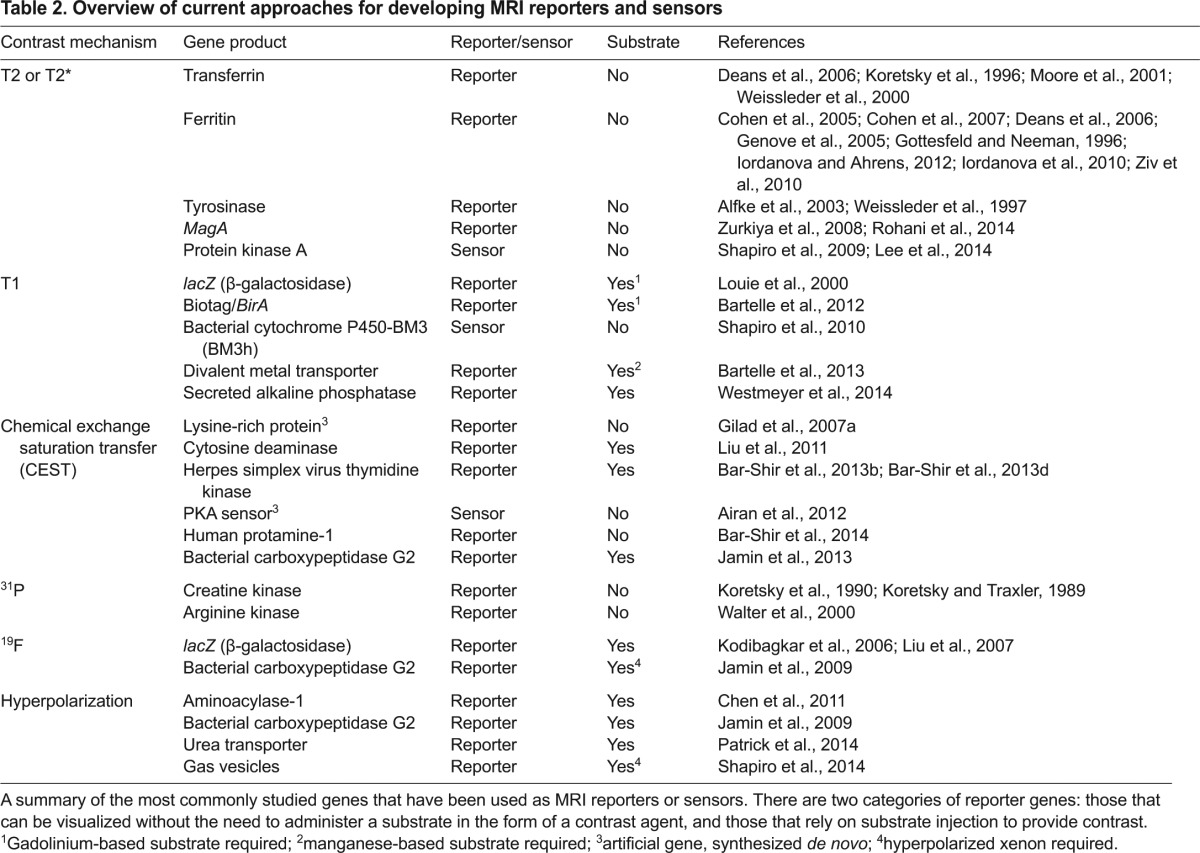
Overview of current approaches for developing MRI reporters and sensors

### Detecting gene expression: use of reporters

With recent advances in the field of molecular imaging, it is now possible to non-invasively monitor transgene expression in cells *in vivo* using genetically encoded reporters. The principle behind this is that a new, foreign gene encoding an MRI reporter protein is introduced into the cell’s genome (either stably or transiently), and is then transcribed and translated to produce a new protein, peptide or enzyme that can affect the MRI contrast in a manner that will enable its detection with MRI. Several landmark studies have utilized such genetically encoded proteins as MRI reporters, including the human transferrin receptor (Koretsky et al., 1996), β-gal (Louie et al., 2000), and proteins that are involved in iron metabolism (Alfke et al., 2003) and storage (Cohen et al., 2005; Cohen et al., 2007; Genove et al., 2005; Zurkiya et al., 2008) (Table 2). An overview of these gene reporter systems has been described in detail in several previous reviews and so is not discussed further here (Airan et al., 2013; Gilad et al., 2007b; Gilad et al., 2008b; Vandsburger et al., 2013).

We have previously demonstrated that the artificial CEST-based lysine-rich protein (LRP) reporter gene can be used to distinguish transplanted rat glioma cells that overexpress the reporter transgene from control cells *in vivo* (Gilad et al., 2007a). Since then, CEST MRI has been used to detect expression of the herpes simplex virus (HSV) type-1 thymidine kinase (tk) (Bar-Shir et al., 2013c), and to sense cellular signaling using a genetically encoded biosensor (Airan et al., 2012). The key advantage here is that the gene is replicated with each cell division, enabling longitudinal MRI tracking of the dynamics of biological processes. At this early stage of development, nearly all of these studies have been merely proof-of-principle and not yet applied in specific disease settings.

**Fig. 5.**
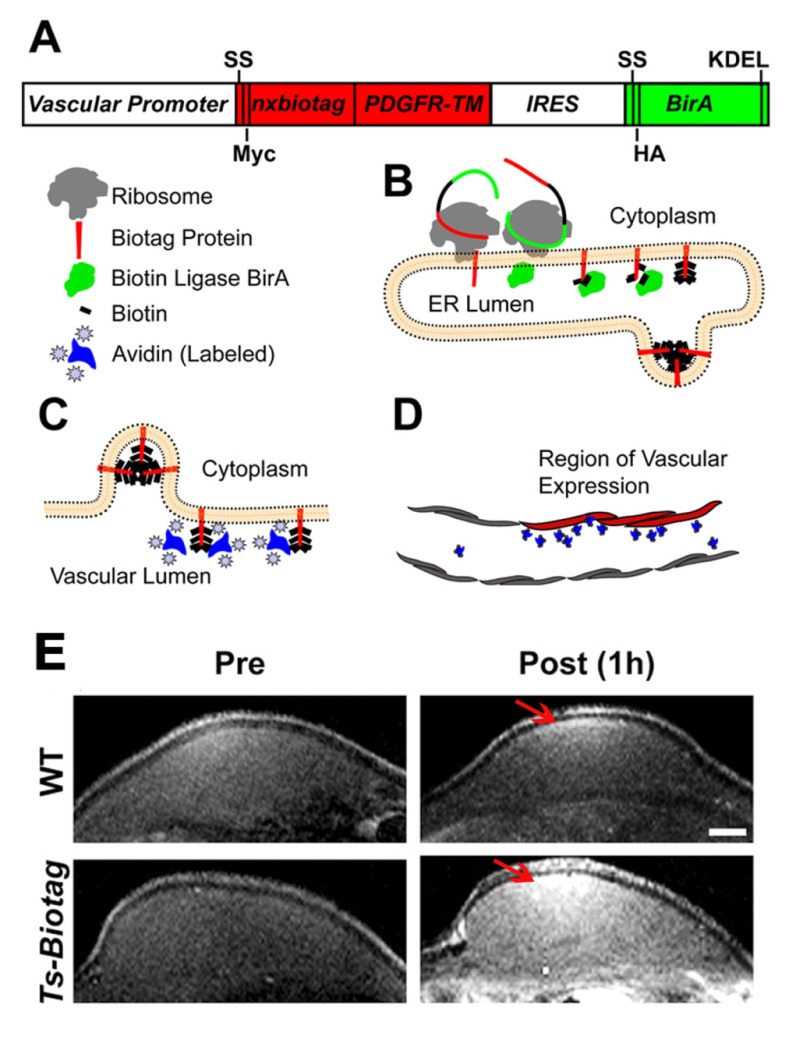
**Schematic of the Biotag reporter system.** The Biotag reporter system, as reported by Bartelle and colleagues (Bartelle et al., 2012), was used here to label endothelial cells in a mouse model of angiogenesis. This system is based on the coexpression and interaction of a Biotag and BirA, and in this study the authors relied on the strong affinity between avidin and biotin. (A) The authors generated transgenic mice that express multiple (nx) copies of a ‘Biotag’ (shown in red). The Biotag was fused to a signal sequence (SS; targeting to the secretory pathway) and the Myc-tagged transmembrane (TM) domain of the platelet-derived growth factor receptor (PDGFR-TM), followed by an internal ribosome entry site (IRES) sequence (allowing translation initiation in the middle of the mRNA sequence) to co-express the hemagglutinin (HA)-tagged BirA enzyme (which is a biotin ligase), modified with SS and KDEL (Lys-Asp-Glu-Leu amino acid) sequences. The expression of this bicistronic construct can be driven by different vascular endothelial cell promoters to restrict its expression to specific cell subtypes. (B) On translation, the ribosome recognizes the N-terminal SS of each protein to insert them into the lumen of the endoplasmic reticulum (ER). In the ER, the BirA enzyme (green) ligates free biotins (black rectangles) onto the Biotag protein (red) and, by continuing through the secretory pathway, the resulting biotinylated Biotag protein will be expressed on the surface of endothelial cells, whereas the BirA enzyme is retained in the ER by the KDEL sequence. (C) Thanks to the strong affinity between biotin and avidin, once on the cell surface, the biotins bound to the Biotag protein will be accessible for binding to the avidinated probes, which can be labeled with fluorescent or paramagnetic agents. (D) Based on the labeling, avidinated probes can be imaged via fluorescence or MR imaging, allowing for the selective detection of the vasculature endothelial cells (red) that express the transgene. (E) To image vascular endothelial cells *in vivo*, transgenic Ts-Biotag mice were generated by using a minimal promoter for tyrosine-protein kinase receptor 2 (T-short; Ts). After injection of DTPA-gadolinium-labeled avidinated probes, Ts-Biotag plugs could be visualized via MRI. Here, *in vivo* MR images of angiogenic endothelial cells within a Matrigel pellet doped with vascular endothelial growth factor, acquired before and 1 hour after injection, are shown (*n*=4). After injection, the immediate vascular tissue close to the plugs is clearly enhanced (arrow) compared with wild type (WT; *n*=4). Figure and data reproduced and modified with permission (Bartelle et al., 2012). Scale bar: 1 mm.

Although there is some overlap between reporter genes and sensors in their function to serve as a beacon, reporters are in general genetically encoded and synthesized by cells, whereas sensors are most often man-made and added to the cells. Importantly, sensors have the capability to sense the extracellular environment and are not limited to act as a beacon for events occurring within or on the surface of the cell. Examples of other recent developments in the field include approaches to express reporters in a specific tissue or cell type [as demonstrated by Bartelle and colleagues, who have taken a creative approach to expressing a multicomponent reporter specifically in endothelial cells (Fig. 5) (Bartelle et al., 2012)]; reporter genes that report on cellular metabolic changes and on the conversion of a pro-drug to an active drug [such as the carboxypeptidase G2 enzyme, a bacterial enzyme with putative therapeutic potential (Jamin et al., 2009)]; and biosensors that detect the activity of proteins, such as PKA (Airan et al., 2012).

### Monitoring cell viability using pH nanosensors

The death of therapeutic cells from immunorejection and/or poor access to oxygen and nutrients during the post-transplantation period is an important factor that reduces the efficacy of stem cell therapies. However, the assessment of cell death after *in vivo* transplantation is challenging. One way to ‘sense’ cell death is to monitor changes in intra- and extracellular pH, a measure of acid-base balance that assesses the effective concentration of hydrogen ions (H^+^). *In vivo*, this acid-base balance is regulated through various pathways that enable the production and transportation of hydrogen ions so that intra- and extracellular pH are physiologically controlled (Gillies et al., 2004). Because of this control, abnormal pH values can indicate cell death or pathological changes in a tissue, e.g. extracellular pH (pHe) is reduced in tumors (Gillies et al., 1994).

Conventional methods that enable the measurement of intra- or extracellular pH *in vitro* include the use of pH electrodes (Roos and Boron, 1981) and the use of optical dyes (Li et al., 2006; Thomas et al., 1979). However, several MRI-based methods that are noninvasive, such as MRS, T1 contrast MRI and CEST contrast MRI, allow pH to be measured in live animals. Our group has recently developed a new technique using pH-sensitive CEST agents to monitor the viability of hepatocytes non-invasively *in vivo* using MRI (Chan et al., 2013). CEST contrast provides an alternative MRI method with which to measure pH (Aime et al., 2002; Liu et al., 2013; Sherry and Woods, 2008; Ward and Balaban, 2000) through changes in the water proton (MRI) signal. This technique offers several advantages; for example, CEST contrast is not constantly ‘turned on’ as T1 relaxation agents are, but is instead switched on through the addition of an appropriate saturation pulse(s) at the start of the imaging sequence, which allows for more-specific detection of contrast resulting from pH changes.

### Imaging of tissue-mimicking scaffolds

**Fig. 6.**
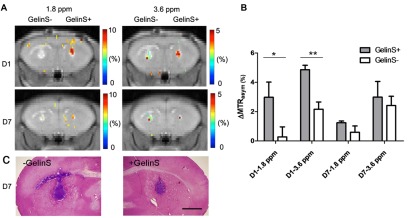
**CEST MRI of gelatin hydrogel scaffold decomposition.** (A) Chemical exchange saturation transfer (CEST) MR images were acquired for hyaluronic acid hydrogel implants with (GelinS+) and without (GelinS–) gelatin at day 1 (D1) and day 7 (D7) post-injection. Hydrogel implants are visible as colored pixels, with the CEST signal indicated by color scale. Left and right panels show CEST images obtained at 1.8 and 3.6 parts per million (ppm) offset from the water proton frequency, respectively. (B) Quantification of CEST contrast in the hydrogel implants shown in panel A. Values shown are the % CEST signal (% MTR_asym_) mean values±s.e.m. (*n*=4). Decomposition of gelation is seen as a decrease in % CEST signal. *P≤0.05; **P≤0.01. (C) Histological assessment at day 7 after transplantation using hematoxylin–eosin–cresyl-violet staining, in which the scaffold appears purple, showing anatomical colocalization with the MRI and proving the origin of the CEST signal. Scale bar: 1 mm. Reproduced with permission (Liang et al., 2015).

As mentioned above, one of the persisting limitations of stem cell transplantation is their poor survival immediately post- transplantation. To this end, tissue-mimicking scaffolds have been used to house and direct cells to grow, with the goal to improve stem cell survival. These scaffolds can be composed of several materials, including hyaluronic acid hydrogels (Liang et al., 2015; Liang et al., 2013b), collagen (Mertens et al., 2014), fibrin (Barsotti et al., 2011) and chitosan (Gomathysankar et al., 2014). The eventual degradation of biomaterial scaffolds is a key factor to successful cell therapy: would the scaffold persist, it might inhibit the integration of regenerated tissue with the host (Zhang et al., 2014). Recently, approaches have been developed to label scaffolds with SPIO and to monitor their site of implantation, tissue integration and biodegradation (Mertens et al., 2014; Mertens et al., 2015). For scaffolds containing gelatin, such as hyaluronic acid hydrogels, a label-free imaging approach has been developed based on its CEST-enhancing properties (Liang et al., 2015) (Fig. 6). It was shown that gelatin decomposition resulted in a reduced CEST contrast, which was validated using fluorescent labeling of the scaffold. Because no label is required, this approach could be of future clinical relevance.

### Metal ion sensing

Metal ions are essential for many biological processes, including for the maintenance of protein structure and for enzymatic activities, as well as in cell signaling and other pathways. The monitoring of metal ion levels is crucial for evaluating the appropriate function and regulation of cellular activity in disease. For example, global alterations in iron levels, including increases in the iron stored by macrophages and microglia, have been observed in the brains of individuals with MS (Stephenson et al., 2014). Similarly, in some neurodegenerative disorders, cellular Ca^2+^ regulation is often compromised, resulting in synaptic dysfunction, impaired plasticity and neuronal degeneration (Mattson, 2007). In ALS, a change in copper (Cu^2+^) homeostasis has been observed (Lovejoy and Guillemin, 2014), which has long been known to occur in Wilson’s disease (Bandmann et al., 2015). In such disorders characterized by irregular metal ion homeostasis, it could be important to evaluate the *in vivo* effect of cell therapy and cell engraftment on metal ion homeostasis as a read-out for therapeutic failure or success.

Although fluorescent-based dyes for imaging metal ions in living cells are commercially available and widely used, their clinical use is limited by the low tissue penetration of the photons they produce. They are, therefore, unsuitable for *in vivo* metal ion imaging in the deep tissues of an intact subject and remain clinically unviable. However, recent advances in the field of molecular MRI have resulted in new strategies for the detection of biologically relevant metal ions that are based on the design and synthesis of responsive contrast agents (Que and Chang, 2010). Efforts have been made to design MRI-responsive agents for the following biologically relevant metal ions: Ca^2+^ (Atanasijevic et al., 2006); Zn^2+^ (Esqueda et al., 2009); Mg^2+^ (Hifumi et al., 2007); Fe^2+^ (Livramento et al., 2005); Cu^2+^ (Que and Chang, 2006); Cu^+^ (Que et al., 2012); and K^+^ (Hifumi et al., 2007). Na^+^ can be observed directly using a coil tuned to the resonance frequency of the naturally occurring isotope ^23^Na (Madelin and Regatte, 2013). MRI sensors for imaging metal ions have been designed to generate MRI contrast in T1 (Li et al., 2002), T2 (Atanasijevic et al., 2006) and CEST (Trokowski et al., 2005) contrast mechanisms; however, only a limited number of studies so far have produced robust *in vivo* data. As an example, it was demonstrated that, by administrating a synthetic MRI probe for sensing Zn^2+^, the functionality of insulin-producing β-cells in healthy mice could be determined *in vivo* (Lubag et al., 2011). Following intravenous glucose injection to stimulate insulin production, the paramagnetic Zn^2+^-responsive T1 agent was administered, resulting in an increase in T1 MRI contrast in healthy mouse pancreas. Because Zn^2+^ release is tightly correlated with insulin release from healthy pancreatic cells, the same experiment performed in diabetic mice (who do not produce sufficient insulin) did not produce any change in MRI contrast. This experiment demonstrates how MRI can reveal cell functionality *in vivo* by detecting changes in metal ion levels, a method that could, in the future, be used to detect changes in insulin release in islet cell transplantation for the treatment of diabetes.

It remains a challenge, however, to image metal ions that are present at low levels with high specificity. To overcome this, we have demonstrated a novel, metal-ion-imaging approach that combines ^19^F MRI with CEST MRI (Bar-Shir et al., 2013a; Bar-Shir et al., 2015) in an approach called (multi)-ion-CEST [(m)iCEST] (see Box 2). Taking advantage of the difference in the ion-specific ^19^F NMR chemical shift offset (Δω) values between the ion-bound and free ^19^F iCEST probe, we exploited the dynamic exchange between ion-bound and free iCEST probe to obtain MRI contrast. It was demonstrated that a prototype single ^19^F iCEST probe could separately visualize mixed Zn^2+^ and Fe^2+^ ions in a specific and simultaneous fashion. A key advantage of this technique is that it allows the detection of low (biologically relevant) concentrations of metal ions by simply reducing the concentration of the ^19^F probe (up to a detectable level) – a feature that is not available for ^1^H MRI-based approaches. Although the iCEST technique has the potential to be used *in vivo*, further studies are required to prove the feasibility of this approach.

## Clinical translation and current limitations

As pointed out, owing to their metal toxicity once de-chelated, it is highly unlikely that manganese, gadolinium and other lanthanides will have opportunities for clinical translation. However, these contrast agents will continue to aid in the assessment of cell transplantation strategies in experimental settings, which are pivotal as proof-of-principle studies. Tracking cells in patients has only been performed so far with SPIO and perfluorocarbons, and this is likely to remain for some time. Both agents have proven to be safe, with the iron in SPIO being metabolized and reused in the normal body iron pool, whereas fluorine is incompatible with any known biochemical reaction in humans, eventually leaving the body as an inert gas compound. Clinical reporter-gene-based cell imaging using the HSV-tk reporter gene has been implemented using PET and an ^18^F-labeled thymidine kinase substrate (Yaghoubi et al., 2009), raising the possibility that similar studies could be done with a CEST MRI substrate (Bar-Shir et al., 2013b; Bar-Shir et al., 2013c). However, there are different regulatory guidelines for using highly sensitive PET probes at a micro-dosing range, as compared to CEST substrates that need to be given at higher doses. Although the PET reporter gene was developed some 20 years ago (Tjuvajev et al., 1995), the CEST reporter gene/sensor field has just begun. CEST agents are based on natural products and do not contain metals; however, they could be involved in immunological or other biological reactions that are at present unknown. Future bio-clearance and dose-toxicity studies will be needed for the further consideration of CEST agents as a clinically viable approach, but so far there have been no indications that clinical translation is prohibited.

## Conclusions and future prospects

MRI-based cell imaging is a versatile technique because it can employ several different types of contrast. It has evolved, in a short period of time, from being a simple cell-tracking approach to an applied method for sensing the physiological state of cells. Except for SPIO- and ^19^F-labeled cell tracking performed clinically, the newer functional sensing and reporter gene approaches are still in their infancy, just entering from the test tube phase into preclinical testing. We anticipate that at least a few of these newer probes will be further developed and become commercially available, increasing their potential user group dramatically and allowing the field to grow. Then, a forward path towards clinical translation could become a bright new path ahead.
